# A Unified 35-Gene Signature for both Subtype Classification and Survival Prediction in Diffuse Large B-Cell Lymphomas

**DOI:** 10.1371/journal.pone.0012726

**Published:** 2010-09-13

**Authors:** Yu-Dong Cai, Tao Huang, Kai-Yan Feng, Lele Hu, Lu Xie

**Affiliations:** 1 Institute of Systems Biology, Shanghai University, Shanghai, People's Republic of China; 2 Key Laboratory of Systems Biology, Shanghai Institutes for Biological Sciences, Chinese Academy of Sciences, Shanghai, People's Republic of China; 3 Shanghai Center for Bioinformation Technology, Shanghai, People's Republic of China; 4 Centre for Computational Systems Biology, Fudan University, Shanghai, People's Republic of China; Indiana University, United States of America

## Abstract

Cancer subtype classification and survival prediction both relate directly to patients' specific treatment plans, making them fundamental medical issues. Although the two factors are interrelated learning problems, most studies tackle each separately. In this paper, expression levels of genes are used for both cancer subtype classification and survival prediction. We considered 350 diffuse large B-cell lymphoma (DLBCL) subjects, taken from four groups of patients (activated B-cell-like subtype dead, activated B-cell-like subtype alive, germinal center B-cell-like subtype dead, and germinal center B-cell-like subtype alive). As classification features, we used 11,271 gene expression levels of each subject. The features were first ranked by mRMR (Maximum Relevance Minimum Redundancy) principle and further selected by IFS (Incremental Feature Selection) procedure. Thirty-five gene signatures were selected after the IFS procedure, and the patients were divided into the above mentioned four groups. These four groups were combined in different ways for subtype prediction and survival prediction, specifically, the activated versus the germinal center and the alive versus the dead. Subtype prediction accuracy of the 35-gene signature was 98.6%. We calculated cumulative survival time of high-risk group and low-risk groups by the Kaplan-Meier method. The log-rank test p-value was 5.98e-08. Our methodology provides a way to study subtype classification and survival prediction simultaneously. Our results suggest that for some diseases, especially cancer, subtype classification may be used to predict survival, and, conversely, survival prediction features may shed light on subtype features.

## Introduction

As the most common subtype of non-Hodgkin lymphomas (NHL), diffuse large B-cell lymphoma (DLBCL) accounts for 30 to 40 percent of lymphoid neoplasm [1]. Diffuse large B-cell lymphoma is an aggressive, fast-growing lymphoma that can arise in lymph nodes or outside of the lymphatic system (e.g., in the gastrointestinal tract, testes, thyroid, or skin). Currently, diagnosis and classification of lymphoma are based on histological recognition of tumor cells complemented by immunophenotyping [2], [3]. The heterogeneous clinical course and different treatment responses within the same diagnostic category, however, suggest that current diagnostic methods should be improved [4]. Identifying patterns of gene expression can foster understanding of the molecular mechanisms of tumorigenesis and allow for the selection of risk-adjusted treatments. Two major subtypes of DLBCL are identified by their genetic activity [5], [6]: activated B-cell-like (ABC) subtype and germinal center B-cell-like (GCB) subtype. We found in the literature several studies of gene expression profiles in DLBCL patients, with some studies focusing on disease subtypes classification [7], [8] and others on survival prediction [9]. As it is known that the GCB subtype has a better prognosis than ABC subtype [5] which suggest that the subtype of DLBCL and survival are intertwined, there should exist a common gene expression signature not only for subtype classification but also for survival prediction.

In this study, the gene expression profiles of 350 DLBCL patients were analyzed. We took 350 samples from four groups (ABC dead, ABC alive, GCB dead, and GCB alive), and assuming the group identity of each test sample was unknown, assigned each to one of the four groups during leave-one-out cross-validation. The features that can best discriminate the four groups of patients were ranked by the mRMR (Maximum Relevance & Minimum Redundancy) [10] principle. Then we applied the IFS (Incremental Feature Selection) procedure to select an optimized feature set. During IFS procedure, each test sample was predicted to fall into one of the four groups using Nearest Neighbor Algorithm (NNA). As a result, 35 features were chosen. This formed a unified gene signature for both subtype classification and survival prediction in diffuse large B-cell lymphomas, by first separating the subjects into four groups and then merging them for the subtype and survival prediction. The subtype prediction accuracy of the 35-gene signature was 98.6%, as evaluated by leave-one-out cross-validation. The predicted high-risk and low-risk patients had significant different overall survival level and the log-rank test p-value was 5.98e-08.

## Methods

### Dataset

The data used in this work were from a lymphoma/leukemia molecular profiling project [11] that included the gene expression profiles and clinical data of 414 patients with newly diagnosed diffuse large-B-cell lymphoma. The data are publicly available at GEO http://www.ncbi.nlm.nih.gov/geo under accession number GSE10846. We excluded from our study patients with unclassified diagnosis. There remained 350 patients, including 73 ABC alive samples, 94 ABC dead samples, 134 GCB alive samples, and 49 GCB dead samples. After averaging the duplicate probes to gene, filtering the low intensity genes and quantile normalization, we obtained the expression profiles of 11,271 genes in 350 DLBCL patients.

### Minimum-Redundancy-Maximum-Relevance (mRMR) feature selection

Minimum-Redundancy-Maximum-Relevance (mRMR) [10] is a widely used method for feature selection. The goal of mRMR is to select the feature subset that can best characterize the statistical property of a target classification variable, with the constraint that these features are mutually as dissimilar to each other as possible, but marginally as similar to the classification variable as possible.

The feature that has maximum relevance with the target variable and minimum redundancy within the features is defined as a “good” feature. Mutual information (MI) is used to describe both relevance and redundancy:
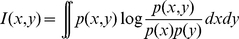
(1)where 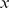 and 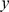 are vectors; 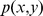 is the joint probabilistic density; 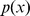 and 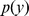 are the marginal probabilistic densities.

The whole vector set is defined as 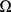, The selected vector set with 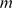 vectors is defined as 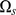, and the to-be-selected vector set with 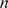 vectors is defined as 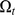. Relevance 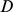 of a feature 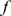 in 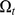 can be calculated by Equation (2):
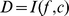
(2)Here 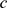 is a classification variable.

Redundancy 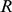 of a feature 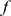 in 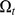 with all the features in 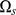 can be calculated by Equation (3):
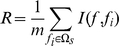
(3)mRMR function maximize relevance and minimize redundancy by integrating Equation (2) and Equation (3):
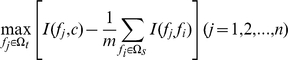
(4)After the pre-evaluation procedure, a feature set 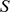 is provided:
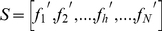
(5)The feature index reflects the evaluations for feature. The feature that fits the Equation (4) better will be added to the set 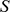 earlier. For example, If a<b, 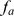 is considered to be better than 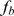.

### Prediction model

With the features selected by mRMR, we used Nearest Neighbor Algorithm (NNA) [12] to classify the samples into the above mentioned categories. NNA predicts a new sample into categories by comparing the features of this sample with the features of those that have known categories. The distance between two vectors 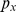 and 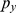 is defined as [13], [14], [15]:
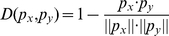
(6)where 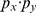 is the inner product of 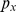 and 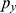, and 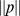 is the module of vector 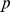. 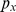 and 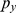 are considered to be more similar if 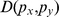 is smaller.

NNA chooses to classify the new pattern 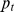 into the class of its nearest neighbor which has the smallest 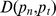. That is:
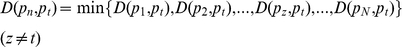
(7)where 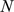 represents the number of training samples.

### Leave-one-out cross-validation method

Leave-one-out cross-validation is an effective and objective way to evaluate prediction performance [14], [15], [16]. Each sample in the data set is knocked out in turn and tested by the predictor trained by the other samples remaining in the data set. During this process, each sample is used not only for the training but also for the testing.

### Evaluation of prediction

Each sample was predicted into one of the groups (ABC dead, ABC alive, GCB dead, or GCB alive), at first. Then the four groups were merged into two classes in two different ways. In subtype classification model, the two classes were activated B-cell-like subtype and germinal center B-cell-like subtype. The predicted ABC subtype samples included the predicted ABC dead and ABC alive samples. The predicted GCB subtype samples included the predicted GCB dead and GCB alive samples. In survival prediction model, the two classes were high-risk group and low-risk group. The predicted high-risk samples included the predicted ABC dead and GCB dead samples. The predicted GCB subtype samples included the predicted ABC alive and GCB alive samples.

To evaluate the performance of subtype classification model, the following equation is used:
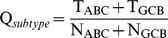
(8)where 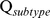 is the overall success rate for subtype prediction. 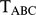 represents the number of corrected predictions for ABC subtype samples, 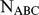 the number of total ABC subtype samples investigated, and so forth.

Cumulative survival time of high-risk samples and low-risk samples was calculated by the Kaplan-Meier method [17] and analyzed by the log-rank test [18]. The log-rank test p-value was used to evaluate the performance of survival prediction model. Statistical analyses were performed by the open-source R software, version 2.10.0 (www.r-project.org).

### Incremental Feature Selection (IFS)

mRMR can only provide a list of features by sorting the features according to their importance to the prediction, but it is still unknown how many fore features in the list should be selected. The best fore features are selected by testing all possible top feature sets and choosing the feature set that can achieve the best prediction accuracy or smallest log rank test p-value. The possible feature subset 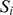 can be expressed using the following equation:
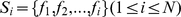
(9)The initial feature subset is 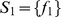, and the last feature subset is 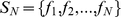 which includes all the features. The leave-one-out test is used to obtain the accurate prediction accuracies of all the feature subsets. The one that can achieve the highest prediction accuracy or smallest log rank test p-value is considered to be the optimized feature set selected by Incremental Feature Selection (IFS) [14], [15], [19]. We can plot a curve, called an IFS curve, with the number of features i as its x-axis and the accurate rate or −log_10_ of the log rank test p-value as its y-axis.

## Results

### mRMR results

Using the mRMR program downloaded from http://penglab.janelia.org/proj/mRMR/, genome-wide 11,271 genes were ranked and the first 500 genes were chosen as potential candidates to discriminate the four groups of patients (ABC dead, ABC alive, GCB dead, and GCB alive). These 500 features are as dissimilar to each other as possible, but as similar to the classification variable as possible.

### IFS results

**Figure 1:**
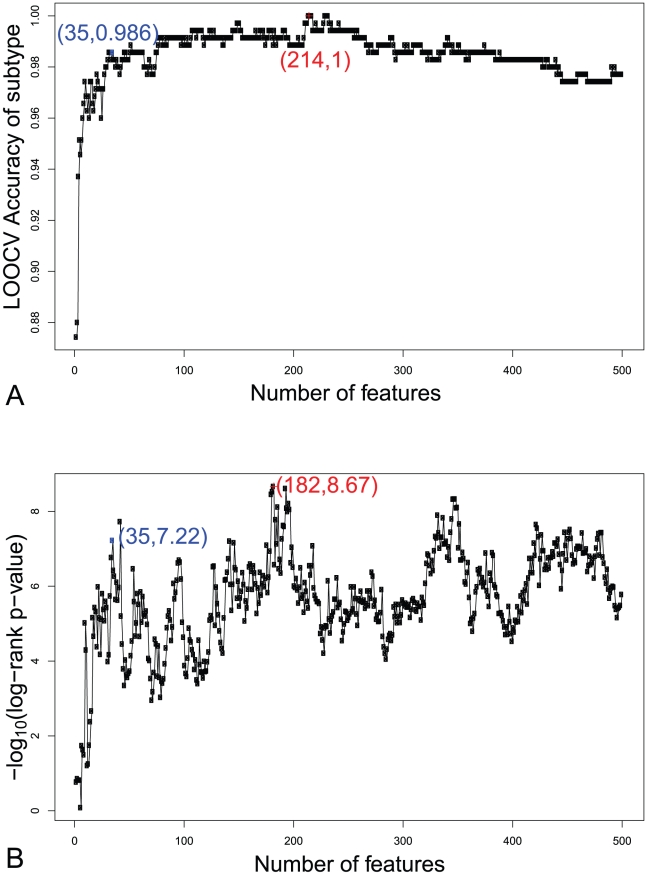
The IFS curves for subtype classification model and survival prediction model. (A) The IFS curve for subtype classification model. The peak overall accuracy was 1 when feature number was 214. However, the overall accuracy had already achieved 0.98 when about 30 features were used. The accuracies only had slight undulation when more features were used. (B) The IFS curve for survival prediction model. The smallest log rank test p-value was 1e- 8.67 when feature number was 182. Local p-values can already reach low when feature number was around 30 to 50. When the optimized 35 features were used the subtype prediction accuracy was 98.6% and the log-rank test p-value was 1e-7.22.

In the IFS procedure, we built 500 feature sets based on the ordered feature set S obtained in the mRMR step. Accordingly, 500 prediction models were constructed and tested as described in the Method section. **Figure 1** shows the IFS curve for (A) subtype classification model and (B) survival prediction model. In the IFS procedure of subtype classification model, the predicted ABC dead and ABC alive samples were combined as predicted ABC subtype samples; the predicted GCB dead and GCB alive samples were combined as the predicted GCB subtype samples. In the IFS procedure of survival prediction model, the predicted ABC dead and GCB dead samples were merged as high-risk samples, and the predicted ABC alive and GCB alive samples were merged as low-risk samples.

In **Figure 1A**, the peak overall accuracy was 1 when the feature number was 214. However, the overall accuracy had already achieved 0.98 when about 30 features were used. The accuracies only had slight undulation when more features were used. In **Figure 1B**, the smallest log rank test p-value was 1e- 8.67 when 182 features were selected. The optimal feature set for subtype classification model and survival prediction model were different, but the fore features were the same.

### Choosing the same feature set for both subtype classification model and survival prediction model

**Figure 2:**
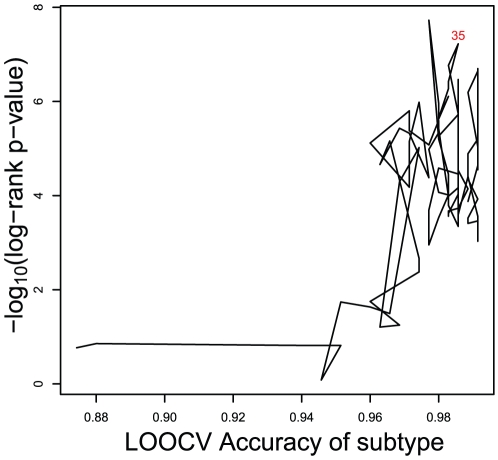
The relationship of subtype classification accuracies and log rank p-values. The x-axis is subtype classification accuracy and the y-axis is −log_10_ of the log rank test p-value. The number of features was restricted to be less than 100 and written on the dot. The number of optimized feature set for both models was 35 which have high subtype classification accuracy and small log rank p-value.

**Figure 3:**
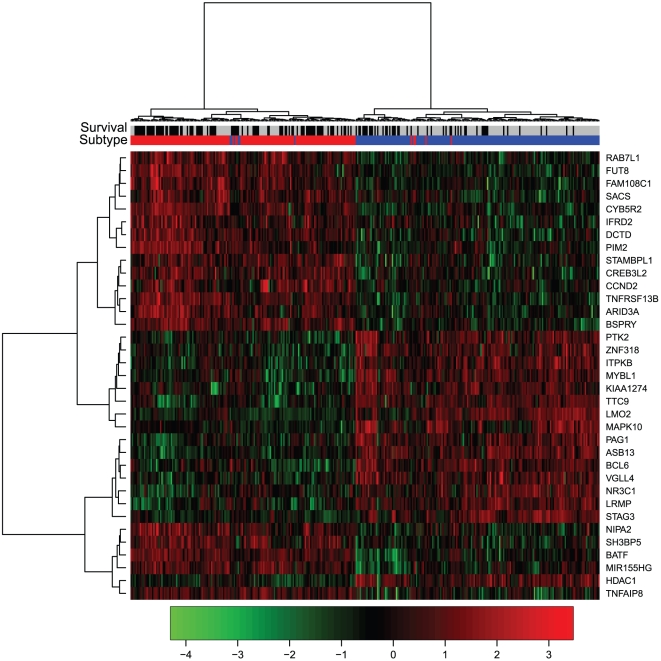
The hierarchical clustering heatmap of patient samples based on expression profiles of the 35-gene signature. Each row represents a signature gene and each column represents a patient sample. The survival and subtype status for each patient are shown with two bars. Black survival bar represents dead, grey survival bar represents alive; red subtype bar stands for ABC subtype, blue subtype bar stands for GCB subtype. The 35-gene signature clearly separated the ABC subtype patients from GCB subtype ones. The dead patients and alive ones were also located at different clusters.

**Figure 4:**
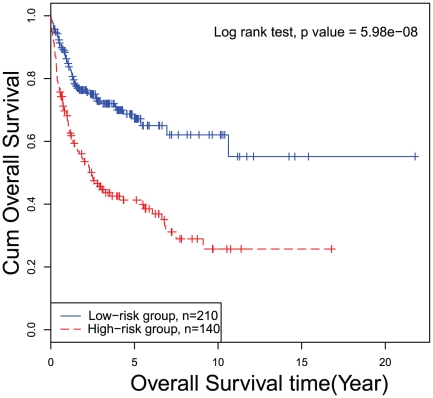
The Kaplan–Meier curve of predicted high-risk and low-risk patients using the 35-gene signature. The log-rank test p-value comparing the overall survival of predicted high-risk and low-risk patients is 5.98e-08.

Although the optimal feature sets for subtype classification model and survival prediction model were not synchronous, we did find a good balance of features for both subtype classification model and survival prediction model, as shown in **Figure 2**. Since subtype classification accuracies increased little when the feature size was larger than 30, and some local minimal p-values were achieved between feature size of 30 and 50, a good, balanced feature set could be chosen with size larger than 30 and less than 50. We investigated the relationship between subtype classification accuracies and log rank p-values by restricting the number of features to less than 100. As shown in **Figure 2**, the size of a proper feature set for both models should be at the top right corner of the plot, indicating both high subtype classification accuracy and small log rank p-value, and it is shown as 35. The subtype prediction accuracy is shown as 98.6%, and log rank p-value is shown as 5.98e-08 (1e-7.22) at the feature set of 35. The unified 35-gene signature for both subtype classification and survival prediction in diffuse large-B-cell lymphomas are given in **Table S1**. The features were sorted according to their importance to the prediction. **Figure 3** shows the hierarchical clustering heatmap of patient samples based on expression profiles of the 35-gene signature. Each row represents a signature gene and each column represents a patient sample. The survival and subtype status for each patient are shown with two bars. Black survival bar represents dead, grey survival bar represents alive; red subtype bar stands for ABC subtype, blue subtype bar stands for GCB subtype. The 35-gene signature clearly separated the ABC subtype patients from GCB subtype ones. The dead patients and alive ones were also located at different clusters. **Figure 4** shows the Kaplan–Meier curve of the predicted high-risk and low-risk patients using the 35-gene signature. The predicted high-risk and low-risk patients had significant different overall survival level and the log-rank test p-value was 5.98e-08.

### Comparison of our signature with reported subtype genes and survival genes

**Figure 5:**
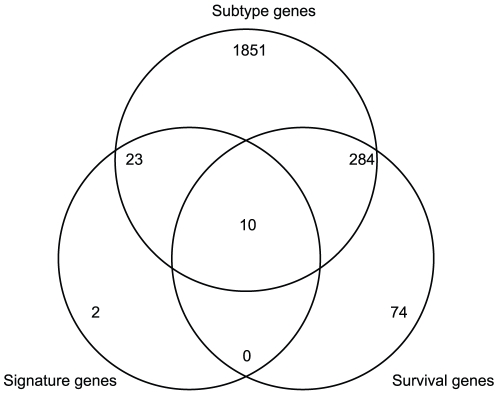
The overlap of our 35-gene signature with reported subtype genes and survival genes. 33 genes from our 35-gene signature are reported to be either subtype genes or survival genes.

We compared our 35-gene signature with reported subtype genes and survival genes. From SignatureDB [20] (http://lymphochip.nih.gov/signaturedb/), we downloaded 16 subtype gene signatures [21], [22], [23] and 7 survival gene signatures [11], [22]. Our 35 genes mapped on to 10 subtype gene signatures and 2 survival gene signatures. **Figure 5** shows the overlap of our 35-gene signature with reported subtype genes and survival genes. Detailed information of each gene in our signature can be found in **Table S1**. It can be seen from **Figure 5** that 33 genes from our 35-gene signature are reported to be either subtype genes or survival genes. There are two genes, NIPA2 and IFRD2, which are not reported as subtype genes or survival genes in SignatureDB. NIPA2 is a selective magnesium transporter [24]. It has been reported that Nipa2 is related with mammary tumorigenesis in mice [25]. IFRD2, interferon-related developmental regulator 2, is a Myc target gene involved in lymphomagenesis [26].

## Discussion

### The biological roles of the 35-gene signature

KEGG enrichment of the 35-gene signature using GATHER [27] (**Table S2**) reveals that the signature genes are related to focal adhesion, cell cycle and Wnt signaling pathway. The enriched KEGG pathways have a close relationship with cancer.

LMO2, MYBL1, BCL6, LRMP, and CCND2 in our 35 signature genes were also reported in Lossos's 36 genes, which predicted survival in diffuse large-B-cell lymphoma [9]. LMO2, ranking second in our signature list, was considered the strongest indicator in Lossos's six-gene signature [9] for survival prediction. MYBL1, ranking third in our list, was also reported in Alizadeh's study of DLBCL subtype classification [6]. According to the mRMR feature list, BCL6 ranked 12th (**Table S1**) and BCL2 ranked 250th (data not shown). GCB subtype is accompanied with a chromosomal translocation involving gene BCL2. The expression of BCL6 may strongly predict survival in patients with diffuse large B-cell lymphoma [28]. CCND2 ranking 35 on our signature list was reported to be the target of BCL6 [9], [29].

A number of other genes ranking high in our 35-gene signature list are functionally important for tumorigenesis. BATF is a basic leucine zipper transcription factor that belongs to AP-1 super family. Stat3 modulates AP-1 activity through the induction of BATF when Stat3 mediates cellular responses associated with proliferation, survival and differentiation [30]. It was reported that in M1 mouse myeloid leukemia cells, forced expression of BATF resulted in a reduced rate of cellular growth [30]. In low grade fibromyxoid sarcoma, a chromosomal aberration involving CREB3L2 was found [31], [32]. In Cancer Gene Census (http://www.sanger.ac.uk/genetics/CGP/Census/), CREB3L2 is recorded as a cancer gene of fibromyxoid sarcoma. Histone deacetylase 1 (HDAC1) is responsible for the deacetylation of lysine residues on the N-terminal part of the core histones [33], [34]. It interacts with tumor-suppressor protein of retinoblastoma [35]. Histone deacetylases play an important role in cell growth arrest, differentiation, and death, generating substantial interest in HDAC inhibitors as possible antineoplastic agents [36]–[39]. PTK2 is a focal adhesion-associated protein kinase implicated in signaling pathways involved in cell motility, proliferation, and apoptosis [40], [41]. It is required for prostate cancer cell motility [42]. PIM2 is a proto-oncogene [43] that acts as a serine/threonine protein kinase. It can prevent apoptosis and to promote cell survival [44], [45], [46].

### The relationship of subtype classification model and survival prediction model

In DLBCL studies, there are two major tasks: subtype classification and survival prediction. Furthermore, they are interrelated (e.g., GCB subtype has a better prognosis than ABC subtype [5]). Knowledge about subtype classification can improve performance on survival prediction, and vice versa. To mutually improve the subtype classification and survival prediction models with the aid of the other, first we divided the samples into four groups and then merged the four groups into two classes in two different ways. A balance of these two models was achieved with the 35-gene signature. The 35-gene signature proved to be useful in both subtype classification and survival prediction of diffuse large-B-cell lymphomas. Our methodology provides a way to study subtype classification and survival prediction simultaneously. Our results suggest that for some diseases, especially cancer, subtype classification may be used to predict survival, and, conversely, survival prediction features may shed light on subtype features.

## Supporting Information

Table S1 The 35 genes in our signature
(0.03 MB XLS)

Table S2 KEGG enrichment of the 35 genes in our signature using GATHER
(0.02 MB XLS)

## References

[1] (1997). A clinical evaluation of the International Lymphoma Study Group classification of non-Hodgkin's lymphoma. The Non-Hodgkin's Lymphoma Classification Project.. Blood.

[2] Veelken H, Vik Dannheim S, Schulte Moenting J, Martens UM, Finke J (2007). Immunophenotype as prognostic factor for diffuse large B-cell lymphoma in patients undergoing clinical risk-adapted therapy.. Ann Oncol.

[3] Berglund M, Thunberg U, Amini RM, Book M, Roos G (2005). Evaluation of immunophenotype in diffuse large B-cell lymphoma and its impact on prognosis.. Mod Pathol.

[4] Lossos IS, Levy R (2003). Diffuse large B-cell lymphoma: insights gained from gene expression profiling.. Int J Hematol.

[5] Turgeon ML (2005). Clinical hematology: theory and procedures.

[6] Alizadeh AA, Eisen MB, Davis RE, Ma C, Lossos IS (2000). Distinct types of diffuse large B-cell lymphoma identified by gene expression profiling.. Nature.

[7] Lenz G, Wright GW, Emre NC, Kohlhammer H, Dave SS (2008). Molecular subtypes of diffuse large B-cell lymphoma arise by distinct genetic pathways.. Proc Natl Acad Sci U S A.

[8] Monti S, Savage KJ, Kutok JL, Feuerhake F, Kurtin P (2005). Molecular profiling of diffuse large B-cell lymphoma identifies robust subtypes including one characterized by host inflammatory response.. Blood.

[9] Lossos IS, Czerwinski DK, Alizadeh AA, Wechser MA, Tibshirani R (2004). Prediction of survival in diffuse large-B-cell lymphoma based on the expression of six genes.. N Engl J Med.

[10] Peng H, Long F, Ding C (2005). Feature selection based on mutual information: criteria of max-dependency, max-relevance, and min-redundancy.. IEEE Trans Pattern Anal Mach Intell.

[11] Lenz G, Wright G, Dave SS, Xiao W, Powell J (2008). Stromal gene signatures in large-B-cell lymphomas.. N Engl J Med.

[12] Friedman JH, Baskett F, Shustek LJ (1975). An algorithm for finding nearest neighbors.. IEEE Trans Comput.

[13] Qian Z, Cai YD, Li Y (2006). A novel computational method to predict transcription factor DNA binding preference.. Biochem Biophys Res Commun.

[14] Huang T, Cui W, Hu L, Feng K, Li YX (2009). Prediction of pharmacological and xenobiotic responses to drugs based on time course gene expression profiles.. PLoS One.

[15] Huang T, Shi XH, Wang P, He Z, Feng KY (2010). Analysis and prediction of the metabolic stability of proteins based on their sequential features, subcellular locations and interaction networks.. PLoS ONE.

[16] Huang T, Tu K, Shyr Y, Wei CC, Xie L (2008). The prediction of interferon treatment effects based on time series microarray gene expression profiles.. J Transl Med.

[17] Andersen PK, Gill RD (1982). Cox's Regression Model for Counting Processes: A Large Sample Study.. The Annals of Statistics.

[18] Harrington DP, Fleming TR (1982). A Class of Rank Test Procedures for Censored Survival-Data.. Biometrika.

[19] Huang T, Wang P, Ye ZQ, Xu H, He Z (2010). Prediction of Deleterious Non-Synonymous SNPs Based on Protein Interaction Network and Hybrid Properties.. PLoS ONE.

[20] Shaffer AL, Wright G, Yang L, Powell J, Ngo V (2006). A library of gene expression signatures to illuminate normal and pathological lymphoid biology.. Immunol Rev.

[21] Wright G, Tan B, Rosenwald A, Hurt EH, Wiestner A (2003). A gene expression-based method to diagnose clinically distinct subgroups of diffuse large B cell lymphoma.. Proc Natl Acad Sci U S A.

[22] Rosenwald A, Wright G, Chan WC, Connors JM, Campo E (2002). The use of molecular profiling to predict survival after chemotherapy for diffuse large-B-cell lymphoma.. N Engl J Med.

[23] Rosenwald A, Wright G, Leroy K, Yu X, Gaulard P (2003). Molecular diagnosis of primary mediastinal B cell lymphoma identifies a clinically favorable subgroup of diffuse large B cell lymphoma related to Hodgkin lymphoma.. J Exp Med.

[24] Goytain A, Hines RM, Quamme GA (2008). Functional characterization of NIPA2, a selective Mg2+ transporter.. Am J Physiol Cell Physiol.

[25] Koch JG, Gu X, Han Y, El-Naggar AK, Olson MV (2007). Mammary tumor modifiers in BALB/cJ mice heterozygous for p53.. Mamm Genome.

[26] Marinkovic D, Marinkovic T, Kokai E, Barth T, Moller P (2004). Identification of novel Myc target genes with a potential role in lymphomagenesis.. Nucleic Acids Res.

[27] Chang JT, Nevins JR (2006). GATHER: a systems approach to interpreting genomic signatures.. Bioinformatics.

[28] Lossos IS, Jones CD, Warnke R, Natkunam Y, Kaizer H (2001). Expression of a single gene, BCL-6, strongly predicts survival in patients with diffuse large B-cell lymphoma.. Blood.

[29] Shaffer AL, Yu X, He Y, Boldrick J, Chan EP (2000). BCL-6 represses genes that function in lymphocyte differentiation, inflammation, and cell cycle control.. Immunity.

[30] Senga T, Iwamoto T, Humphrey SE, Yokota T, Taparowsky EJ (2002). Stat3-dependent induction of BATF in M1 mouse myeloid leukemia cells.. Oncogene.

[31] Storlazzi CT, Mertens F, Nascimento A, Isaksson M, Wejde J (2003). Fusion of the FUS and BBF2H7 genes in low grade fibromyxoid sarcoma.. Hum Mol Genet.

[32] Panagopoulos I, Storlazzi CT, Fletcher CD, Fletcher JA, Nascimento A (2004). The chimeric FUS/CREB3l2 gene is specific for low-grade fibromyxoid sarcoma.. Genes Chromosomes Cancer.

[33] Ammanamanchi S, Freeman JW, Brattain MG (2003). Acetylated sp3 is a transcriptional activator.. J Biol Chem.

[34] Hung JJ, Wang YT, Chang WC (2006). Sp1 deacetylation induced by phorbol ester recruits p300 to activate 12(S)-lipoxygenase gene transcription.. Mol Cell Biol.

[35] Nicolas E, Morales V, Magnaghi-Jaulin L, Harel-Bellan A, Richard-Foy H (2000). RbAp48 belongs to the histone deacetylase complex that associates with the retinoblastoma protein.. J Biol Chem.

[36] Choi JH, Kwon HJ, Yoon BI, Kim JH, Han SU (2001). Expression profile of histone deacetylase 1 in gastric cancer tissues.. Jpn J Cancer Res.

[37] Zhang Z, Yamashita H, Toyama T, Sugiura H, Ando Y (2005). Quantitation of HDAC1 mRNA expression in invasive carcinoma of the breast*.. Breast Cancer Res Treat.

[38] Toh Y, Yamamoto M, Endo K, Ikeda Y, Baba H (2003). Histone H4 acetylation and histone deacetylase 1 expression in esophageal squamous cell carcinoma.. Oncol Rep.

[39] Noonan EJ, Place RF, Pookot D, Basak S, Whitson JM (2009). miR-449a targets HDAC-1 and induces growth arrest in prostate cancer.. Oncogene.

[40] Kallergi G, Agelaki S, Markomanolaki H, Georgoulias V, Stournaras C (2007). Activation of FAK/PI3K/Rac1 signaling controls actin reorganization and inhibits cell motility in human cancer cells.. Cell Physiol Biochem.

[41] Shim H, Lee H, Jeoung D (2006). Cancer/testis antigen cancer-associated gene (CAGE) promotes motility of cancer cells through activation of focal adhesion kinase (FAK).. Biotechnol Lett.

[42] Lacoste J, Aprikian AG, Chevalier S (2005). Focal adhesion kinase is required for bombesin-induced prostate cancer cell motility.. Mol Cell Endocrinol.

[43] Baytel D, Shalom S, Madgar I, Weissenberg R, Don J (1998). The human Pim-2 proto-oncogene and its testicular expression.. Biochim Biophys Acta.

[44] Dai H, Li R, Wheeler T, Diaz de Vivar A, Frolov A (2005). Pim-2 upregulation: biological implications associated with disease progression and perinueral invasion in prostate cancer.. Prostate.

[45] Gong J, Wang J, Ren K, Liu C, Li B (2009). Serine/threonine kinase Pim-2 promotes liver tumorigenesis induction through mediating survival and preventing apoptosis of liver cell.. J Surg Res.

[46] Yan B, Zemskova M, Holder S, Chin V, Kraft A (2003). The PIM-2 kinase phosphorylates BAD on serine 112 and reverses BAD-induced cell death.. J Biol Chem.

